# Editorial: Women's Under-representation in Engineering and Computing: Fresh Perspectives on a Complex Problem

**DOI:** 10.3389/fpsyg.2018.00595

**Published:** 2018-04-24

**Authors:** Kathleen Buse

**Affiliations:** Case Western Reserve University, Cleveland, OH, United States

**Keywords:** women in STEM, engineering, computing, women, under-representation

Understanding the many complexities that define gender inequality has been described by researchers as a grand challenge (Joshi et al., 2015). Novel insights, innovation, a broader community to conduct research and to ascertain effective interventions are essential in the challenge to create organizations that are gender equal. As such, this research topic in Frontiers in Psychology addresses the underrepresentation of women in engineering and computing as a complex, but solvable problem. The special issue seeks to inform the global community about advances in understanding the underrepresentation of women in engineering and computing with a focus on what enables change. Further this issue promotes fresh perspectives, innovative methodologies, and mixed method approaches important to accelerating the pace of change.

Despite more than 40 years of research attempting to explain the gender gap (Kanny et al., 2014) the engineering and computing professions continue to be dominated by men. In 2015 women comprised 12% of the engineers and 25% of computer professionals in the USA (US Bureau of Labor Statistics, 2015). This is at a time when women comprise 47% of the total labor force and 52% of managers and professionals. The numbers are similar in Europe, India, and Australia, but slightly higher in China (The Association of German Engineers, 2010; Catalyst, 2012, 2015; Engineers Australia, 2017).

An intentional, international effort is essential to accelerate the rate of achievement for women in engineering and computing. As such this special research topic includes 13 papers that employ empirical methodologies studying adolescents, students in 2-year colleges, undergraduate and graduate programs, and women in the workplace. The full body of work includes studies related to career choice, academic success, workplace recruitment, retention, and advancement. Methodologies employed include qualitative, quantitative, longitudinal, ethnographic, case studies, and mixed methods. The papers span the development of theoretical concepts through effective interventions and suggestions on future research.

Several of the contributions leverage empirical methods to describe interventions. Adolescents are the focus of the longitudinal study by Riegle-Crumb and Morton that explores the impact of messages from peers within science classrooms on girls' career choice in the science, technology, engineering, and math (STEM) professions. Results reveal that a higher percentage of 8th grade male peers in the classroom who endorsed explicit gender/STEM stereotypes significantly and negatively predicted girls' later intentions to pursue engineering or computer science. Yet results also reveal that exposure to a higher percentage of confident female peers in the science classroom positively predicted such intentions. These results were specific to engineering and computer science, suggesting that peers are an important source of messages regarding girls' career choice to pursue non-traditional STEM fields.

Women's educational pathways and success in STEM through 2-year colleges were explored by Wang et al. Specifically they studied the relationship between the intent to transfer and a set of motivational, contextual, and socio-demographic background factors. Six hundred and ninety-six female students from 2-year colleges in the USA were surveyed as they began studies in STEM programs or courses. The survey data and administrative records were used to complete a multinomial logistic regression analysis. Findings revealed that students' math and science self-efficacy beliefs, as well as transfer-oriented interaction, were significant and positive predictors for their intent to transfer into STEM fields. The association between transfer intent and motivational and contextual factors was moderated by students' racial/ethnic backgrounds, marital status, and childcare obligations. These findings have important and nuanced implications for policymakers, educators, and researchers and support the case for women to consider 2-year colleges as pathways to success in STEM fields.

An innovative approach was used by Milesi et al. employing Experience Sampling Method (ESM) to evaluate, in real-time, the engagement of college students. 165 students majoring in computer science at two Research I universities were “beeped” several times a day via a smartphone app prompting them to provide information. The responses were paired with data provided by the institutions. Mean comparisons and logistic regression analysis were used to compare enrollment and persistence patterns. Results suggest that women are more likely to continue taking computer science courses when they felt challenged and skilled in their initial computer science classes. This promising study was the first using this method. The authors suggest expanding the study across different types of institutions and over a longer time period for further insight on the relationship between engagement and persistence.

Some German universities offer separate women's engineering courses which include virtual STEM learning environments. Christophel and Schnotz explore the virtual learning environments and whether there are differences in the virtual learning of female and male learners in STEM courses. A field study was conducted with 56 students (female = 27, male = 29) completing a virtual STEM learning program. Results revealed that strategic competences were positively correlated with situational interest in the virtual learning environment regardless of gender. In contrast, the correlations between mental effort and competences differed between female and male participants. Female learners' mental effort decreased if they had more strategic competences while female learners' mental effort increased if they had more arithmetic-operative competences. The finding that female learners are more likely than their male colleagues to be influenced by strategic and arithmetic-operative competences regarding their mental effort indicates that separate women's courses may be an effective approach for increasing their representation in engineering.

The “Grad Cohort,” a multi-day mentorship workshop for women graduate students developed by the Computing Research Association's Committee on the Status of Women in Computing Research (CRA-W), is an intervention strategy studied by Stout et al. The long-term impact of this program on the Ph.D. students' professional goals, computing identity, and entity beliefs was studied and compared to a sample of women and men Ph.D. students in computing programs who had never participated. Grad Cohort participants reported interest in becoming well-known in their field to a greater degree than women non-participants, and to an equivalent degree as men. Also, Grad Cohort participants reported stronger interest in giving back to the community than their peers. Further, whereas women non-participants identified with computing to a lesser degree than men and held stronger entity beliefs than men, Grad Cohort participants' computing identity and entity beliefs were equivalent to men. Importantly, stronger entity beliefs predicted a weaker computing identity among students, with the exception of Grad Cohort participants. This latter finding suggests Grad Cohort may shield students' computing identity from the damaging nature of entity beliefs. Together, these findings suggest Grad Cohort may fortify women's commitment to pursuing computing research careers and move the needle toward greater gender diversity in computing.

Photovoice, a Participatory Action Research method, was utilized by Amon to identify themes that underlie women's experiences in traditionally male-dominated fields. Photovoice enables participants to convey unique aspects of their experiences via photographs and their in-depth knowledge of a community through personal narrative. Forty-six STEM women graduate students and postdoctoral fellows from a large university in the USA participated in the Photovoice activity in small groups. They presented photographs that described their experiences pursuing leadership positions in STEM fields. Findings provide a basis for better understanding the complex ways that gender stereotypes surface in organizations, as well as the bottom-up strategies STEM women employ to cope with workplace challenges. Specifically, results provide a framework for understanding women's work preferences, challenges, and buffering strategies. This work generated insight into policies that can best support and enhance STEM women's career success including explicitly advocating for workplace collegiality, offering structured networking opportunities, instituting faculty mentorship programs along with mentorship training, and using incentives to increase departmental and interdepartmental collaboration.

Carrigan offers an ethnographic study of women's experiences in computing providing evidence of an existing systemic preference for the technical dimensions of computing over the social and a correlation between gender and social aspirations. The study suggests there is a gap between the current state of computing and the yearning of participants who desire to use computing to contribute to the collective well-being of society. Findings from the study suggest that gender equity in computing may be achieved by addressing the meaning of the social values, ideologies and practices of these institutions. A call for the development of measures and evaluations on how computing contributions to society is made along with a call for further studies in this area.

Fouad et al. utilized qualitative analysis on a national sample of 1,464 women engineers to identify the reasons women leave the profession. The findings show that women's decisions to leave their jobs and the engineering field was shaped by a lack of fit between their needs and values and the reinforcements present in their work environment. Further the results show that occupational values and needs related to comfort, safety, and achievement predominantly characterized women engineers' attrition decisions. Occupational reinforcers include status, altruism, and autonomy needs. Their detailed understanding helps promote organizational interventions aimed at engaging and retaining women in the profession. Specifically interventions should provide career development opportunities for women engineers. Work should include opportunities that allow a sense of accomplishment, security, good compensation, opportunities for advancement, and a workplace free from discrimination and harassment.

New theoretical directions are needed that provide a framework for continued understanding of the underrepresentation of women. Expanding current conceptual frameworks to better understand the underrepresentation of women in engineering and computing is important. Steinke does this while focusing on the role of popular media in the formation of adolescent girls' STEM identity. Steinke argues that the media plays a crucial role in the construction, representation, reproduction, and transmission of STEM stereotypes and that this may be particularly salient and relevant for girls during adolescence as they actively consider future personal and professional identities. Gender-stereotyped media images of STEM professionals are described and theories are examined to identify variables that explain the potential influence of these images on STEM identity formation. This article emphasizes the importance of focusing on STEM identity relevant variables and STEM identity status to explain individual differences in STEM identity formation.

A new framework for continued understanding of the underrepresentation of women in engineering and computing is provided by Buse et al. Arguing that an extensive body of research on gender equity already exists, this paper details a future research agenda that was developed from dialogue among 50 experts at a 2 day convening. The result is an innovative, collaborative approach to future research that focuses on identifying effective interventions. The new approach includes the creation of partnerships with stakeholders including businesses, government agencies, non-profits, and academic institutions to allow a broader voice in setting research priorities. Researchers recommend incorporating multiple disciplines and methodologies, while expanding the use of data analytics, merging and mining existing databases, and creating new datasets. Studies are recommended that focus on socio-cultural interventions particularly on career choice, within undergraduate and graduate programs, and for women in professional careers all leading to gender equity in the engineering and computing professions.

Critical reviews of the literature specifically those that identify gaps in understanding of women's experiences in engineering and computing including explanations of why these gaps exist and how to increase understanding are important to this topic. In a focused review, Boucher et al. discuss how stereotypic perceptions of computing and engineering influence who enters, stays, and excels in these fields. The focus here is on communal goal incongruity defined as the idea that some disciplines (like engineering and computing) are perceived as less aligned with people's communal goals of collaboration and helping others. Empirical literature is reviewed that demonstrates how perceptions of these disciplines are incongruent with communal goals and how this perception deters women and girls. This perspective is extended by reviewing accumulating evidence that perceived communal goal incongruity can deter any individual who values communal goals. The authors conclude that communal opportunities within computing and engineering have the potential to benefit first generation college students, underrepresented minority students, and communally-oriented men (as well as communally-oriented women). The implications is that those opting out of STEM perpetuate the stereotypic (mis)perceptions of computing and engineering. Recommendations are presented to highlight communal opportunities in computing and engineering to increase interest and motivation. By better integrating and publically acknowledging communal opportunities, the stereotypic perceptions of these fields could gradually change, making computing and engineering more inclusive and welcoming to all.

Case studies describing interventions that have improved the representation of women in engineering and/or computing, with an emphasis on the psychological and organizational factors that impacted women's ability to achieve within the organizations are important to this topic. A case study of a research-based approach to retaining and advancing women in the STEM professions is detailed by Van Oosten et al. The Leadership Lab for Women is an innovative professional development program that leverages recent research, 360° feedback, coaching, and practical strategies. The program provides women with knowledge, tools, and a supportive learning environment to help them navigate, achieve, flourish, and catalyze organizational change in male-dominated and technology-driven organizations. Early analysis of the program's outcomes show increases in participant's self-awareness, self-efficacy, and ability to persist, and excel in their chosen profession. These provide encouraging signs of the intervention's success in advancing and retaining women.

As one part of a multi-phase case study spanning over a decade Crenshaw et al. add to the understanding of the cultural and experiential issues impacting the retention of women and people in the computing disciplines. Their study compares data gathered at the Department of Computer Science at the University of Illinois at Urbana-Champaign in 2005 and more recently in a follow-up project completed in 2016. The data reveals improvements in the perceptions of undergraduate teaching quality and undergraduate peer mentoring networks. However, evidence was found of continuing feelings of isolation, incidents of bias, policy opacity, and uneven policy implementation particularly with respect to historically underrepresented groups. Preliminary findings, research and methodological reflections, and applied research suggestions are presented that aim to create positive cultural change in computing.

In closing we want to thank the many authors, reviewers, and editors who contributed to this special research topic addressing the underrepresentation of women in engineering and computing. You have worked collaboratively to inform the global community not only about advances in understanding the underrepresentation but have specified many intervention strategies that enable change. The grand challenge of gender equity in engineering and computing will be overcome as researchers, practitioners, and educators continue to work together to develop knowledge and apply intervention strategies to accelerate the rate of change.

## Author contributions

The author confirms being the sole contributor of this work and approved it for publication.

### Conflict of interest statement

The author declares that the research was conducted in the absence of any commercial or financial relationships that could be construed as a potential conflict of interest. The handling Editor declared a shared affiliation, though no other collaboration, with the author.
